# Inequities in energy-balance related behaviours and family environmental determinants in European children: baseline results of the prospective EPHE evaluation study

**DOI:** 10.1186/s12889-015-2540-5

**Published:** 2015-12-02

**Authors:** Krystallia Mantziki, Achilleas Vassilopoulos, Gabriella Radulian, Jean-Michel Borys, Hugues Du Plessis, Maria João Gregório, Pedro Graça, Stefaan De Henauw, Svetoslav Handjiev, Tommy LS Visscher, Jacob C Seidell

**Affiliations:** Department of Health Sciences, VU University Amsterdam, Amsterdam, The Netherlands; Department of Agricultural Economics and Rural Development, Agricultural University of Athens, Athens, Greece; “Carol Davila” University of Medicine and Pharmacy, Bucharest, Romania; EPODE European Network Coordinating Team, Proteines, Paris, France; Faculty of Nutrition and Food Sciences, University of Porto, Porto, Portugal; Directorate General of Health, Lisbon, Portugal; Department of Public Health, Ghent University, Ghent, Belgium; Bulgarian Association for the study of Obesity and related diseases, Sofia, Bulgaria; Research Centre for the Prevention of Overweight, Windesheim University of Applied Sciences Zwolle & VU University, Zwolle, The Netherlands

**Keywords:** Health inequalities, Childhood obesity, Dietary intake, Screen exposure, Family environmental determinants, EPODE

## Abstract

**Background:**

Tackling inequalities in overweight, obesity and related determinants has become a top priority for the European research and policy agendas. Although it has been established that such inequalities accumulate from early childhood onward, they have not been studied extensively in children. The current article discusses the results of an explorative analysis for the identification of inequalities in behaviours and their determinants between groups with high and low socio-economic status.

**Methods:**

This study is part of the Epode for the Promotion of Health Equity (EPHE) evaluation study, the overall aim of which is to assess the impact and sustainability of EPODE methodology to diminish inequalities in childhood obesity and overweight. Seven community-based programmes from different European countries (Belgium, Bulgaria, France, Greece, Portugal, Romania, The Netherlands) participate in the EPHE study. In each of the communities, children aged 6–8 years participated, resulting in a total sample of 1266 children and their families. A parental self-administrated questionnaire was disseminated in order to assess the socio-economic status of the household, selected energy balance-related behaviours (1. fruit and vegetable consumption; 2. soft drink/ fruit juices and water consumption; 3. screen time and 4. sleep duration) of the children and associated family environmental determinants. The Mann–Whitney U test and Pearson’s chi-square test were used to test differences between the low and high education groups. The country-specific median was chosen as the cut-off point to determine the educational level, given the different average educational level in every country.

**Results:**

Children with mothers of relatively high educational level consumed fruits and vegetables more frequently than their peers of low socio-economic status. The latter group of children had a higher intake of fruit juices and/or soft drinks and had higher screen time. Parental rules and home availability were consistently different between the two socio-economic groups in our study in all countries. However we did not find a common pattern for all behaviours and the variability across the countries was large.

**Conclusions:**

Our findings are indicative of socio-economic inequalities in our samples, although the variability across the countries was large. The effectiveness of interventions aimed at chancing parental rules and behaviour on health inequalities should be studied.

**Electronic supplementary material:**

The online version of this article (doi:10.1186/s12889-015-2540-5) contains supplementary material, which is available to authorized users.

## Background

Over the past 20 years, numerous studies have examined social differences in lifestyle, in an effort to explain social inequalities in health [1]. Nowadays it is established that pronounced socio-economic inequalities-defined by the educational level and/or occupational class and/or income- in non-communicable diseases exist between and within countries in Europe [2–8], even at the local level, namely within cities, communities and neighbourhoods [3, 4, 6, 8–10]. Recent evidence shows that obesity rates are higher and are growing more rapidly in populations with relatively low socio-economic status [4, 5, 7, 8, 11–16], while socio-economic inequalities in obesity are broadening in the European region [11]. In addition, it is well-established that individuals of middle and lower income, occupation class and/or educational level are more likely to develop non-communicable diseases and to be more exposed to related risk factors [2–5, 7, 9, 11, 15]. This may, at least partly, be explained by relatively unhealthy dietary habits and a less active lifestyle which are more common amongst subgroups with a relatively low socio-economic status [6, 7, 10, 11, 17–22].

Inequalities in childhood obesity and overweight have not been studied extensively. Robertson et *al.* report in their review that there is a general an association between parental socio-economic status and the prevalence of obesity and overweight in European children [7]. A more recent study, however, found variations in socio-economic disparities regarding childhood overweight across European regions, suggesting the need for further research in nationally representative samples [23]. At the local level, data show that particular neighbourhoods have both increased rates of childhood overweight as well as unhealthy behaviour [10] and that there are associations between lower family income/parental education with increased childhood obesity rates [15, 22]. Additionally, findings from the Health Survey of England showed that despite the levelling-off of childhood obesity and overweight prevalence between 2004–2007, the socio-economic disparities were have increased [24].

Tackling inequalities in overweight, obesity and related determinants has become a top priority for the European research and policy agendas over the last few years [5, 7, 8, 11, 25]. Based on the fact that such inequalities accumulate from early childhood onward [3, 26] and that childhood is a critical period for shaping behaviours, targeting children is of major importance. Nevertheless, evidence for the effectiveness of interventions in reducing inequalities in obesity and overweight in children are scarce [4, 7, 16, 25]. Research into the socio-economic differences in behaviours and determinants of behaviours across different populations could give insight into what kinds of interventions are needed to successfully decrease socio-economic inequalities.

The current study aims to identify the differences in energy balance-related behaviours and explore related environmental determinants, between high and low socio-economic groups. Specifically, it will provide evidence for inequalities in unhealthy behaviours and related determinants, in different urban populations from cities across seven European countries.

## Design and methods

This study is part of the EPHE (Epode for the Promotion of Health Equity) evaluation study [27], the overall aim of which is to assess the impact and sustainability of the EPODE (Ensemble Prévenons l’Obésité Des Enfants-Together let’s prevent obesity) methodology [28, 29] in diminishing inequalities in childhood obesity and overweight. Here we present and describe the results of the baseline measurements.

It is a two-year follow up study, that seeks to identify inequalities in energy-balance related behaviours (EBRB) of children and their related family-environmental determinants, while also assessing the effectiveness and sustainability of EPODE methodology to change those behaviours and determinants in populations from low socio-economic status [27]. The current paper presents the baseline measurements, which are results of a descriptive and explorative analysis for the identification of inequalities in behaviours and their determinants between groups of high and low socio-economic status.

The survey obtained formal declaration from the Medical Ethics Committee of the VU University Medical Centre, that it does not fall under the scope of the Medical Sciences people research Act (WMO). In addition, permission to research in schools was acquired from local community and/or school authorities, where necessary.

### Sample and recruitment

**Fig. 1:**
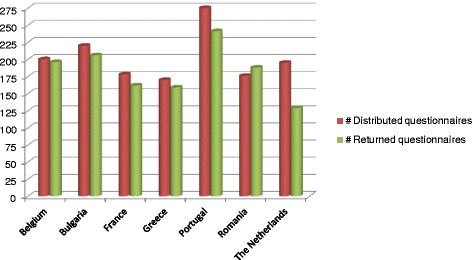
Number of invited and recruited children to the EPHE baseline measurements per country

Seven community-based programmes which are part of the Epode International Network and implement the EPODE methodology participate in the EPHE project: VIASANO (Belgium), EPODE (France), PAIDEIATROFI (Greece), Maia Healthy Menu (Portugal), SETS (Romania), JOGG (The Netherlands) HEALTHY KIDS (Bulgaria); the latter programme is part of the Nestle’s Healthy Kids programme and implements similar methodology to EPODE. Every programme is based in a medium-sized city. We aimed at recruiting a minimum of 150 families with children aged between 6 to 8 years old in every selected community with a similar variation regarding age and ethnicity per site. The recruitment was conducted through schools. More information about sampling and recruitment are described elsewhere [27]. The number of invited and finally recruited children is indicated in Fig. 1.

### Data collection

The questionnaires, including an informed consent, were distributed to the children at school and delivered to their parents, between May/June 2013, before the intervention period. After a specified period of one to two weeks, the completed questionnaires were collected and only the ones including a signed informed consent were taken into consideration. In order to ensure the confidentiality of the data, a process to guarantee anonymity of participant families was applied [27].

### Development of the EPHE parental questionnaire

In order to identify inequalities, i.e. socio-economic differences in energy-balance related behaviours and their determinants, a self-administered parental questionnaire was constructed. The EPHE parental questionnaire was developed using items from relevant, validated questionnaires addressed in European populations: ENERGY parent and child questionnaires [30], the Pro-children child questionnaire [31] and its updated version PRO-GREENS [32], European Health Examination Survey questionnaire [33], European Social Survey questionnaire [34], United States Department of Agriculture questionnaire [35]. Additional items were constructed since no validated items or questionnaires existed to our knowledge. The rationale and development of the questionnaire are described in detail elsewhere [27].

### Assessment of energy-balance related behaviours in the EPHE parental questionnaire

The questionnaire assessed four energy-balance related behaviours of the child: 1. fruit and vegetable consumption; 2. soft drink/fruit juices and water consumption; 3. screen time and 4. sleep duration, as well as determinants related to the social and physical environment of the child, within the family setting. In order to keep the length of the questionnaire within acceptable limits, we had to prioritise the many aspects of behaviour that could be relevant. The Scientific Committee decided (in consultation of experts) to keep sedentary behaviour as the indicator of physical activity. Other relevant aspects which were not included were snacks and meals (such as breakfast, lunch and dinner) and consumption of energy-dense food .

The consumption of fruits and vegetables was assessed by food frequency questions, referring to a usual week and measured in an 8-point Likert scale (1.Never-8.Every day, more than twice a day) [32]. The consumption of fruit juices, soft drinks and diet soft drinks was measured by means of weekly frequency and amount consumed. The frequency was measured in a 7-point Likert scale (1.Never-7.Every day, more than once a day) [30]. The amount was measured by 2 items for fruit juices and 3 items for soft and diet soft drinks, assessing how many glasses (or small bottles; 250 ml), cans (330 ml) or big bottles (500 ml) the children drink [30]. The amount was calculated by summing the portions. In order to measure water consumption two questions were constructed to measure the daily frequency (1.Never-7. More than 6 times a day) and number of glasses consumed when drinking water (1. None-6. 5 or more glasses). Sedentary behaviour is assessed by means of daily time spent in television (TV) viewing and time of computer (PC) use, for the week and the weekend days separately, measured in a 9-point Likert scale (1.Not at all-9. 4.0 or more hours a day) [30]. The total screen time was calculated by the sum of weekly (hours per weekday*5 + hours per weekend day*2) TV and PC use. Furthermore, 2 questions informed by the ENERGY parent questionnaire assess the sleeping habits of the child (1.sleeping routine; 2.sleep duration per week/weekend-day) [30].

### Assessment of determinants of energy-balance related behaviours in the EPHE parental questionnaire

The determinants assessed refer to the social and physical family environment of the child. These were mainly assessed by one item and most of them measured in a 5-point Likert-types scales (0. never - 4. always or −2. fully disagree - 2. fully agree), unless otherwise stated below and in the tables of this article. The social environmental determinants are: a. for *fruit and vegetable* consumption i. parental demand (0. never - 4. yes, always), ii. parental allowance (0. never - 4. yes, always), iii. active encouragement (−2. fully disagree - 2. fully agree), iv. facilitating 0. never - 4. yes, always) and v. parental knowledge on recommendations (1. no fruit – 8. 5 pieces per day) [32]; b. for *fruit juice\soft drink* consumption and *TV viewing\computer* exposure i. paying attention\monitoring (0. never - 4. always), ii. parental allowance (0. never - 4. always), iii. Negotiating (0. never - 4. always), iv. communicating health beliefs (0. never - 4. always), v. avoid negative modelling (0. never - 4. always), vi. parental self-efficacy to manage child’s intake (0. never - 4. always), vii. rewarding\comforting practice (0. never - 4. always), viii. conducting energy-balance related behaviour together with the child (1. Never- 8. Every day more than once; for TV viewing/computer time the scale is “0. never - 4. always”) [32]. The physical environmental determinants are: a. for the consumption of *fruit and vegetables* i. home availability (0. never – 4. always) and ii. situation specific habit (−2. fully disagree - 2. fully agree) b. for *fruit juices/soft drinks* consumption i. home availability (0. never - 4. yes, always) and ii. situation specific habit (1. yes - 2. no); c. for *water* consumption i. situation specific habit- measured by three items (0. never - 4. always) and d. for *TV viewing\computerexposure *i. availability (1.yes - 2.no) ii. situation specific habit (1. every day – 5. never) more details are described in Mantziki et al. [27].

### Socio-economic assessment

Socio-demographic characteristics (Table 1) were measured in a. Likert-type scales (i. age of the respondent: 1. 20 and below-6. 41 and above; ii. age of the child: 1.6 years olds- 4. 9 years old and above; iii. parental education level: 1. Less than 6 years-6. More than 17 years; iv. perception of income: 1. Living comfortable in the present income-4. Finding it difficult in present income), b. in 8-category scale (i. labour status; ii. source of income), c. in 6-category scale (sector of employment). The food security level of the household was also assessed [27].

**Table 1:** Socio-demographic characteristics of the EPHE population per country

Country	Total n	Gender		Age child (years)	Age of mother^a^		Income position^b^		Employment status mother		Educational level mother	
		Boys (%)	Girls (%)	Mean (SD)	<30 (%)	>30 (%)	Good (%)	Not good (%)	Employed (%)	Not employed (%)	High (%)	Low (%)
Belgium	196	53,4	45,4	6,58 (0,55)	21,4	77,9	88,8	11,2	64,8	24,5	42,7	57,3
Bulgaria	205	46,8	52,7	7,97 (0,78)	8,7	90,1	81,8	18,2	84,1	15,9	74,3	25,7
France	160	38,8	57,5	6,34 (0,55)	30,9	69,1	79,6	20,4	53,5	46,5	35,2	64,8
Greece	159	46,5	45,9	7,37 (0,66)	3,2	94,4	51,0	49,0	61,5	38,5	52,8	47,2
Portugal	241	51,0	48,5	6,85 (0,74)	12,4	87,1	55,8	44,2	73,8	26,2	46,0	54,0
Romania	176	56,8	43,2	7,39 (0,54)	17,7	82,3	75,9	24,1	78,0	22,0	53,8	46,2
The Netherlands	129	47,3	52,7	7,83 (0,98)	6,5	90,7	87,9	12,1	76,8	21,4	61,3	38,7
Total	1266	49,8	49,2	7,17 (0,90)	14,6	84,4	73,6	26,4	72,5	27,5	52,7	47,3

^a^The analysis includes the age of the mother only when the mother was the respondent; the age of the second parent was not assessed. Response categories: 1 = Below 20, 2 = 21–24, 3 = 25–30, 4 = 31–35, 5 = 36–40, 7 = Above 40. Number of subjects included in “age of mother” per country: Belgium = 148, Bulgaria = 171, France = 136, Greece = 128, Portugal = 208, Romania = 147, The Netherlands = 107, Total = 1038

^b^Income position categories: (1) Living comfortably on present income (2) Coping on present income (3) Finding it difficult on present income (4) Finding it very difficult on present income. Income position was defined as “good” when the response was (1) or (2) and “not good” when the response was (3) or (4)

Two socio-economic groups were distinguished, based on classification for each indicator assessed: “mother’s and father’s employment status” (employed - not employed), “income position” (good – not good), “mother’s and father’s educational level” (low-high). The aforementioned variables are described in detail by Mantziki et *al*. Subdivision into two socio-economic groups was very unequal when based on employment status and income position for the majority of the samples (Table 1). In addition, knowing that educational level has been classified as a good social factor that explains differences in nutritional outcomes [1, 20, 23], for the current article, the samples were divided in two groups based on the “educational level of the mother” (low-high). For each country’s sample the median of the educational level was used as the cut-off point to determine the educational level of the mother (low-high).

### Statistical analysis

All the datasets were checked for missing and double-crossed (more than one boxes selected in an item, either by mistake or because the answer was between 2 categories) values. The double-crossed values were corrected where possible, by choosing the valid selection or selecting the more frequent of the two options selected. The total sample analyses included all subjects from all communities. Due to minor discrepancies between the translated versions of the questionnaire, i.e. missing response categories in certain items, minor adaptations in the response categories were made when necessary.

The Mann–Whitney U test for the ordinal variables and Pearson’s Chi-square test for the binary variables were used to detect differences in behaviours and determinants between the two socio-economic groups. Here we present medians and quartile ranges (Mann–Whitney U test), as well as percentages (Pearson’s Chi-square) in order to illustrate the differences between the two groups. Knowing that the mean ranks produced by non-parametric tests are not always sufficiently informative and that differences in spread may be equally important as differences in medians [36], further assessment of frequencies and distributions per item was explored. The results of the additional assessments are discussed in the article, but not presented for practical reasons.

All analyses were conducted using the SPSS software 21.0 (IBM Corp., Armonk, NY, USA).

Adjustment for multiple testing was conducted using the Benjamini and Hochberg method [37], using the Stata software 13 (StataCorp. 2013. *Stata Statistical Software: Release 13*. College Station, TX: StataCorp LP).

## Results

A total of 1266 children and their families were included in the EPHE study. Table 1 summarizes the socio-demographic characteristics of the population per country. In all counties boys and girls represented almost 50 % each of the recruited samples and the average age of the participant children was 7 years old. The response rates per country were more than 85 % for all countries, excluding the Netherlands where the response rate was 65 % (Fig. 1).

Given the large variation of identified differences per country, in this paper we focus on discussing the statistically significant differences in the samples.

### Inequalities in energy balance-related behaviours

Children of the high education groups consumed fruit significantly more frequently during the week than their peers from the low education group (Table 2). Vegetable consumption was also higher for some high education groups, while the same trend was observed for the overall sample for both fruit and vegetable consumption (Table 2).

**Table 2:** Rounded median values and quartiles (q_1_-q_3_) for weekly dietary intake for each educational group per country

	Fruit consumption (frequency/week)^a^		Salad/grated vegetables consumption (frequency/week )^a^		Raw vegetables consumption (frequency/week )^a^		Cooked vegetables consumption (frequency/week)^a^	
Educational level	High	Low	High	Low	High	Low	High	Low
Country								
Belgium	4 (4–6)	4 (3–5)	3 (2–4)	4 (2–5)	3 (2–5)	3 (2–4)	5 (4–6)	4 (4–6)
Bulgaria	6 (4–6)	6 (4–6)	5 (4–6)	5 (4–6)	5 (4–6)	5 (4–6)	4 (3–5)	4 (4–5)
France	4 (3–6)	4 (3–5)	4 (2–5)	4 (2–4)	4 (2–4)	3 (2–4)	4 (3–6)	4 (3–5)
Greece	6 (4–6)	6 (4–6)	4 (4–6)	5 (4–6)	3 (2–4)*	**4 (3–4)**	4 (3–6)	3 (3–4)
Portugal	7 (6–7) ***	**6 (5–7)**	6 (5–7)***	**5 (4–6)**	4 (3–5)	4 (2–5)	7 (6–7)***	**6 (5–7)**
Romania	6 (4–6)*	**5 (4–6)**	4 (4–6)	4 (4–5)	4 (3–5)	4 (3–5)	5 (4–6)*	**4 (4–6)**
The Netherlands	6 (5–7)**	**5 (4–6)**	4 (3–4)	4 (3–4)	3 (2–4)	4 (3–4)	5 (4–5)	5 (4–5)
Total	6 (4–7)***	**5 (4–6)**	4 (3–6)*	**4 (3–6)**	4 (3–5)**	**4 (2–4)**	5 (4–6)	4 (4–6)

Comparison between the educational groups of each country and the total sample with Mann–Whitney U test.

*,**,***: significant at .05, .01 and .001 respectively

^a^Response categories: **1**.Never **2**.Less than one day per week **3.**One day per week **4**.2-4 days a week **5**.5-6 days a week **6.**Every day, once a day **7.**Every day, twice a day **8.**Every day, more than twice a day

Differences between the high and low education groups were also observed in the amount and of fruit juices and soft drinks (Table 3) consumed on a weekly basis. The values demonstrate that children with mothers of low education in all countries were more likely to have a higher amount (in ml) of intake when they drank fruit juices/soft drinks; though statistical significance varied at country-level and was not found in all countries. Results from the total participating population indicate the same trends for the amount of fruit juices/ soft drinks consumed and for the frequency of soft drinks consumption (Table 3). With regard to the frequency of fruit juices we observed that in some communities it was higher in the high education group compared to the low education group, while in the most of them the opposite was observed (Table 3). Water consumption frequency was significantly higher for the low education group in two of the communities, whereas no difference was found in the rest of them.

**Table 3:** Rounded median values and quartiles (q_1_-q_3_) for weekly beverage intake for each educational group per country

	Fruit juices frequency^a^		Fruit juices amount (ml)^c^		Soft drinks frequency^a^		Soft drinks amount (ml)^c^		Water frequency^b^	
Educational level	High	Low	High	Low	High	Low	High	Low	High	Low
Country										
Belgium	6 (4–6)	6 (4–7)	500 (250–580)*	**580 (250–750)**	4 (2–5)	4 (2–5)	250 (250–580)	500 (250–580)	4 (3–5)	4 (4–5)
Bulgaria	4 (3–5)	4 (3–5)	580 (500–830)*	**830 (580–1160)**	2 (1–3)	2 (2–4)	250 (0–958)	500 (250–750)	5 (5–6)**	**6 (5–6)**
France	4 (4–6)	6 (4–6)	250 (250–790)	580 (250–830)	3 (2–4)	4 (2–5)	330 (250–580)	580 (250–1020)	5 (4–6)	4 (4–5)
Greece	4 (4–6)	4 (4–5)	580 (250–580)	580 (580–830)	1 (1–2)	2 (1–2)	250 (0–393)	250 (0–580)	5 (4–6)**	**5 (5–6)**
Portugal	4 (2–4)	4 (3–4)	580 (250–580)	580 (250–580)	2 (1–3)**	**2 (2–3)**	250 (250–580)*	**500 (250–580)**	5 (4–6)	5 (4–6)
Romania	4 (3–5)*	**4 (2–4)**	580 (250–830)*	**580 (580–1160)**	2 (1–3)***	**3 (2–4)**	580 (62,5-580)***	**830 (330–1080)**	5 (5–6)	5 (5–6)
The Netherlands	4 (2–5)	3 (2–5)	375 (250–580)*	**580 (250–1000)**	4 (2–6)	3 (2–6)	250 (250–580)	250 (250–750)	3 (3–4)	4 (3–4)
Total	4 (4–6)	4 (3–6)	580 (250–580)***	**580 (250–830)**	2 (1–4)***	**3 (2–4)**	250 (0–580)***	**580 (250–750)**	5 (4–6)	5 (4–6)

Comparison between the educational groups of each country and the total sample with Mann–Whitney U test.

*,**,***: significant at .05, .01 and .001 respectively

^a^Response categories: **1**.Never **2**.Less than once a week **3.**Once a week **4**.2-4 days a week **5**.5-6 days a week **6.**Every day, once a day **7.**Every day, more than once a day

^b^Response categories: **1**.Never **2**.Less than once per day **3.**Once a day **4**.2-4 times a day **5**.5-6 times a day **6.** More than 6 times a day

^c^The indicated amounts are derived from the sum of the respective question items; J3a and J3b and K3a, K3b and K3c for fruit juices amount and soft drinks amount respectively (27). The variables are categorical with specific values of ml in each category

Furthermore, for the children of the low education group in all countries higher amounts of screen time were reported, with a statistically significant difference between the two groups in the majority of the participant countries (Table 4). A noteworthy finding is the amount of time spent watching TV during the week, which was higher for the low education group in all countries and the difference with the high education group reached statistical significance in almost all countries. Similar were the differences regarding the time spent watching TV in weekend days, reaching statistical significance in some of the samples (Table 4). Computer time was significantly higher for the low education group in a few samples during weekdays and weekend days as well. Consistent results were observed in the total sample; children of the low education group in all countries spent more time in front of screens (total screen time) during the week than their counterparts of the high education group (Table 4). There was also disparity between the groups in terms of sleep duration only in two countries (Table 4). We were unable to identify significant differences between the education groups for sleep duration in the total sample.

**Table 4:** Rounded median values and quartiles (q_1_-q_3_) for screen exposure and sleep hours per educational group per country

	TV weekdays (h/day) ^a^		TV weekend days (h/day) ^a^		PC weekdays (h/day) ^a^		PC weekend days (h/day) ^a^		Total screen time (h/week) ^c^		Sleep duration weekdays (h/day) ^b^		Sleep duration weekend days (h/day)^b^	
Educational level	High	Low	High	Low	High	Low	High	Low	High	Low	High	Low	High	Low
Country														
Belgium^d^	3 (2–4)***	**4 (3–6)**	5 (4–7)**	**7 (5–9)**	1 (1–2)**	**2 (1–3)**	2 (2–4)	3 (1–5)	12,5 (9,0 -18,0)***	**19,0 (12,0-26,0)**	3 (2–3)	3 (2–3)	3 (3–3)	3 (2–3)
Bulgaria	3 (3–4)	4 (3–5)	5 (4–7)	6 (4–7)	2 (2–3)***	**3 (2–4)**	3 (2–4)*	**4 (3–5)**	18 (12,4-27)*	**25,7 (14,2-31,2)**	2 (2–2)	2 (2–2)	3 (2–3)	3 (2–3)
France	3 (2–4)**	**4 (3–5)**	5 (4–7)*	**6 (4–8)**	2 (1–2)	2 (1–3)	2 (1–3)	3 (2–4)	14,0 (8,0-24,0)*	**19,5 (11,0-25,0)**	3 (2–3)	3 (2–3)	3 (3–3)	3 (2–3)
Greece	3 (2–4)**	**4 (3–4)**	5 (4–6)	6 (4–7)	2 (1–2)	2 (1–3)	3 (2–4)	3 (2–3)	14,0 (10,0-22,5)*	**18 (13,0-22,5)**	2 (2–3)	2 (2–3)	3 (2–3)	3 (2–3)
Portugal^e^	3 (2–4)**	**3 (3–4)**	5 (4–6)*	**6 (4–7)**	2 (1–2)	2 (1–2)	3 (2–5)	3 (2–5)	14,5 (10,0-20,0)**	**17,5 (12,0-24,5)**	3 (2–3)**	**2 (2–3)**	3 (2–3)	3 (2–3)
Romania^e^	3 (3–5)***	**4 (3–6)**	5 (4–6)	6 (4–7)	2 (1–4)	2 (1–3)	4 (2–5)*	**3 (1–5)**	20 (14,0-25,0)	22,0 (15,0-30,5)	2 (2–3)	2 (2–2)	3 (2–3)	3 (2–3)
The Netherlands^e^	3 (2–4)	3 (3–4)	4 (4–5)	4 (4–6)	2 (2–3)	2 (2–3)	3 (2–4)	3 (2–4)	13,5 (11,0-20,0)	14,5 (11,0-23,5)	3 (3–3)***	**3 (2–3)**	3 (3–3)*	**3 (2–3)**
Total	3 (3–4)***	**4 (3–5)**	5 (4–6)***	**6 (4–7)**	2 (1–3)	2 (1–3)	3 (2–4)	3 (2–5)	15,5 (10–23)***	**19,5 (12,5-25,5)**	2 (2–3)	2 (2–3)	3 (2–3)	3 (2–3)

Comparison between the educational groups of each country and the total sample with Mann–Whitney U test.

*,**,***: significant at .05, .01 and .001 respectively

^a^Response categories: **1.**Not at all **2.**30 min/day **3.1 h/day 4.**2 h/day **5.**2,5 h/day **6.**3 h/day **7.**3,5 h/day **8.**4 or more h/day

^b^Response categories: **1.** 6 hours or less/ per night **2.**7 hours/ per night **3.**8 hours/ per night **4.**9 hours/ per night **5.**10 hours/ per night **6.**More than 10 hours per night.

^c^The indicated amounts of hours are derived from the sum of the respective question items for TV (T1a and T1b) and PC time (T4a and T4b) (27). The variables are categorical with specific values of hours in each category.

^d^the variables TV/PC time for weekdays and weekend-days are measured with an extra response category for 1,5 h/day (coded as 4); as such the items include 9 response categories. This does not apply for the results of the total sample

^e^the variables PC time for weekdays and weekend-days are measured with an extra response category for 1,5 h/day (coded as 4); as such the items include 9 response categories. This does not apply for the results of the total sample

### Inequalities in determinants of fruit and vegetable consumption

Social environment (Additional file 1)*:* Parental demand for fruit consumption was significantly higher for high educated mothers only in one country. Parental allowance for fruit and vegetable consumption was higher for high educated mothers compared to the low educated mothers in one country and for fruit consumption, in the total sample as well. Furthermore, high educated parents from one country reported to eat fruit more frequently with their children (perform energy-balance related behaviour together), than the parents of the respective low education group. In addition, parents of the high education group in some countries served vegetables at meal time (parental facilitation) significantly more frequently, compared to the respective low education groups.

In the overall sample, similar differences in the social environmental determinants of fruit consumption between the low and high education groups were found for parental demand, parental allowance and facilitation of fruit consumption (Additional file 1). Likewise all parental practices related to vegetable consumption, apart from parental demand, were significantly better for the high education group.

Physical environment (Additional file 1): Fruit availability at home was more frequent for children of the high education group of some countries and similarly for the total sample. Availability of vegetables at home was higher for the high education group in only one country compared to the low education group. The same trend was observed in the total sample analysis for the home availability of both fruits and vegetables. Moreover, only in one country children of highly educated mothers were more likely to have the habit of eating vegetables daily, rather than their peers of low educated mothers. This was also observed in the total sample.

### Inequalities in determinants of fruit juices' and soft drinks’ consumption

Social environment: Low educated mothers reported to reward/comfort their child by giving fruit juices more often than high educated mothers, which was the case for the total sample as well (Additional file 2). Additionally*,* in some of our samples parental efficacy to retain rules with regard to the child’s fruit juices’ intake was significantly more frequent in the high educated mothers compared to the efficacy of the low educated mothers (Additional file 2). At the same time, higher frequency of trying to drink fruit juices when intake was prohibited (nagging) was reported for children of low educated mothers (Additional file 2).

In reference to soft drinks’ consumption, more frequent parental allowance was reported by the low educated mothers compared to highly educated mothers in one country and total sample (Additional file 3). In addition, low educated mothers were drinking soft drinks together with their child (perform energy-balance related behaviour together) significantly more often than the highly educated ones, while only one sample of highly educated mothers reported higher frequency of avoiding negative modelling for soft drinks intake (Additional file 3). Nagging for soft drinks’ intake was more frequent for children of some low education groups, compare to the respective higher education groups. In the total sample it was observed that the low educated mothers drank soft drinks together with their child more often compared to the high educated ones (Additional file 3).

Physical environment (Additional file 5): availability of soft drinks at home was more frequent for the children of the low educated groups. Moreover, in the total sample, children of low educated mothers were more likely to drink fruit juices while watching television and soft drinks during the weekend , at lunch and at dinner. The corresponding differences for the situations of habitual intake -both soft drinks and fruit juices-, varied highly across the countries.

### Inequalities in determinants of screen exposure

Social environment (Additional file 4): Highly educated mothers monitored (paying attention/monitoring) the amount of time their child watched television more frequently than the low educated ones. Low educated mothers allowed their children to watch television (parental allowance) more often than the high educated ones, whereas only one sample of highly educated mothers was more likely to restrain watching television in presence of the child (avoid negative modelling) than the low educated mothers. The majority of the low educated groups reported watching television with their children more frequently than the respective high educated groups, although statistical significance varied.

In reference to the social determinants of computer exposure, the highly educated mothers were more likely to negotiate with their child about the time that was allowed to spend on computer activities, compared to the low educated ones. However, the high educated mothers of only one country were more likely to avoid computer use in the presence of their child. Furthermore, children from the low education group were more likely to try playing computer games when it was forbidden (nagging), compared to their peers form the high education group. Parents with low education reported playing computer games together with their child more frequently than the ones with high education (Additional file 4).

Some of the parental practices related to television viewing were more favourable for the high education group in the total sample: parental allowance; parental monitoring; avoiding negative modelling. For the two latter determinants the same trend was observed in reference to computer exposure (Additional file 4).

Physical environment: The majority of low education groups, including the total sample, reported having the television on during meal time significantly more frequently than the high education group. More children of low educated mothers had television in their bedroom than their peers of highly educated mothers. This difference was significant in almost all countries and in the total sample (Additional file 5).

### Results after multiple testing adjustments

Adjustments for multiple testing resulted in critical *p*-values lower than 0.05, as initially set by the authors (Additional file 6). Consequently, less of the differences found within the education groups of each of the samples (based on α = 0.05) were significant based on the adjusted lower threshold (Additional file 6). As an illustration, the statistically significant differences between the two groups in the total sample analysis were initially 44 and after the adjustments these were 41.

## Discussion

This study showed that children from communities of seven different European countries of relatively high socio-economic status consumed fruits and/or vegetables more frequently than their peers of low socio-economic status. In addition, the latter group of children had a higher intake of fruit juices and/or soft drinks and had higher screen time. It is important to note that increased screen activity found among children from lower socio-economic status is attributed to television watching, rather than computer activity.

The results of our study are compatible with studies that demonstrate that children from lower socio-economic status across Europe have unhealthier dietary habits and increased sedentary behaviour compared to their high socio-economic status peers. Norwegian children of lower socio-economic status reported a particularly low frequency of fruit consumption [17]. Furthermore, low vegetable consumption was associated with overweight in Dutch children of lower socio-economic status [30]. The IDEFICS study illustrated that the “healthy” dietary pattern (including fruit and vegetable intake) was positively associated with high socio-economic status, whereas the “processed” pattern (including sweetened drinks) was inversely associated with high socio-economic status [19]. The Healthy Behaviour in School-aged Children (HBSC) study revealed higher fruit consumption for children from high socio-economic status (measured in terms of both the family affluence scale and parental occupation) and higher soft drink consumption with decreasing score of parental occupational class [38]. Elinder et *al.*, found that Swedish children of parents with a relatively low level of education were eating less vegetables and were consuming more sweetened drinks than their peers with highly educated parents [22]. Less is known for socio-economic differences in fruit juices’ consumption among children, although evidence shows higher consumption of fruit juices in children and adolescents living in low-income households in the USA [39]. With respect to television viewing and computer activity, Fairclouhg et *al.* found an inverse association with socio-economic status in 9–10 year-olds [40] and Fernandez-Alvira et *al.* showed that these behaviours partly mediate the association between parental education and child’s body composition [41].

Important differences between the two socio-economic groups in our samples were observed in the determinants of the social and physical family-environment of the child. Despite that we did not find a common pattern for all behaviours, parental rules and home availability were consistently different between the two socio-economic groups in our study in all countries. This indicates the importance of the family environment, related to socio-economic inequalities in childhood obesity. In addition, these differences varied to a large extent across countries, illustrating the heterogeneity of inequalities across the EPHE communities, as other studies also confirm [23, 38].

Family-environmental determinants have been associated with energy-balance related behaviours, although little is known about socio-economic disparities in these associations. A survey reviewing multi-disciplinary literature to identify the determinants of childhood obesity, concluded, among others, that *the shared environment created by parents, affects children’s choices and eventually their body weight outcomes* [42]. Related evidence demonstrates that parental rules and/or accessibility at home are significantly associated with energy balance- related behaviours, such as screen time, intake of sugary drinks and fruit and vegetable consumption [22, 43, 44]. The increased accessibility of fruits and vegetables-measured in terms of home availability, parental facilitation and allowance- have been shown to mediate adolescents’ intake [21, 45]. On the other hand, the presence of screens in the child’s bedroom is associated with higher adiposity in preadolescents [46], while it contributes to the excess of the screen time [47]. Accordingly, we consider that parental rules and home availability are crucial to be addressed in interventions aiming to decrease inequalities in childhood obesity.

Overall, the differences in energy balance-related behaviours and family-related determinants assessed in this study were statistically significant but not large. The significant differences can be explained by *differences in spread* in the response categories of the assessed variables as well as by *differences in the median* and quartile values that are presented in this paper. The Mann–Whitney U test is able to detect differences in shape and spread, which are, usually, equally important as differences in median [36]. Differences in spread could also explain the significant findings when identical median and quartile values were found in both groups. That is to say that the low socio-economic groups were more likely to fall into the less favourable response categories, in the vast majority of the variables assessed, unless otherwise stated in the tables and appendices.

To our knowledge, this is the first evaluation study that provides baseline data on socio-economic inequalities in family-environmental determinants associated with energy-balance related behaviours. The cross-cultural character of the sample enables the exploration of inequalities in factors that have been highly associated with childhood obesity, across different European countries. Hence the opportunity to enhance insight of health inequalities is given, particularly in the European region where the socio-economic factors are changing rapidly over time. Also there is the prospect to sensitize communities with respect to socio-economic inequalities in childhood obesity and overweight. In addition, our results give new insight into energy-balance behaviours and their determinants, which should be the focus for the development of effective interventions aimed at reducing inequalities in childhood obesity. Another strength of this study is the high response rate achieved in almost all countries and successful commitment of the target groups.

For the purposes of the EPHE evaluation study, the participant programmes were selected on the basis of implementing the EPODE or EPODE-like methodology. At this point it should be clarified that the interventions implemented within the EPHE project will be new and specifically focused at the selected behaviours and determinants to reduce health inequalities. Similar to the programme selection, it was a prerequisite for the participant city to be already engaged in an EPODE structure. The schools from which the samples were recruited were selected based on accessibility and convenience criteria. These schools were also chosen due to a limited time-frame. Hence, one limitation of this study is that sampling bias is likely present at many levels and our samples may not be representative of each country’s population. Another weakness of this study could be that we used the educational level of the mother as a proxy for socio-economic status, instead of using more indicators. Although, parental education level has been characterised as an adequate socio-economic indicator by relevant and more elaborative studies [1, 20, 23], this still reduces the strength of detecting absolute inequalities. Moreover, the data were self-reported and recall bias and/or socially desirable answers are possible. Furthermore, errors from the constructed items are possible, given that they were not validated. Another source of bias of our whole-sample results could be from errors in the translated versions of the questionnaires, where, despite efforts regarding forth-back translations, slightly different answer categories were used. This occurred in the variables assessing screen exposure (missing category) and the frequency that the television was on during meal times. Considering that the family environmental correlates are assessed mostly by one item each, the reliability of the instrument may be violated [30]. Finally, this is an observational study and thus conclusions about causality cannot be drawn.

### Implications for public health

In this study we confirm that socio-economic inequalities exist in energy-balance related behaviors in various European communities. Addressing these behaviors may aid in reducing socio-economic differences in health. Moreover, this study has additionally identified community-specific inequalities in the determinants of these behaviors. Targeting these behavioral determinants in public health interventions, aimed at changing these behaviors, in a favorable way may increase their effectiveness.

## Conclusion

Our study indicates socio-economic inequalities in factors strongly related to childhood obesity and overweight and provides evidence for those in seven European communities. These findings are indicative of socio-economic inequalities in our samples, but the variability across the countries was large. The effectiveness of interventions aimed at chancing parental rules and behaviours on health inequalities should be studied.

## Additional files

Additional file 1:
**Median values and quartiles **(q_1_-q_3_)** for determinants of the child’s environment and fruit/vegetable consumption.** (DOCX 25 kb) Additional file 2:
**Median values and quartiles **(q_1_-q_3_)** for determinants of the child’s social environment and fruit juices consumption.** (DOCX 21 kb) Additional file 3:
**Median values and quartiles **(q_1_-q_3_)** for determinants of the child’s social environment and soft drinks consumption**. (DOCX 20 kb) Additional file 4:
**Median values and quartiles **(q_1_-q_3_)** for determinants of the child’s social environment and screen exposure.** (DOCX 27 kb) Additional file 5:
**Median values and quartiles **(q_1_-q_3_)** for determinants of the child’s physical environment and energy-balance related behaviours.** (DOCX 34 kb) Additional file 6:
**Corrected critical** ***p*** **-values after adjustment for multiple testing.** (DOCX 28 kb)

## Abbreviations

EPODE: Ensemble Prévenons l’Obésité Des Enfants-Together let’s prevent obesity
EPHE: EPODE for the promotion of health equity
PC: personal computer
