# Supplementary material for: Inequities in energy-balance related behaviours and family environmental determinants in European children: baseline results of the prospective EPHE evaluation study
Source: BMC Public Health. 2015 Dec 2;15:1203. doi: 10.1186/s12889-015-2540-5 (PMC4668694; doi:10.1186/s12889-015-2540-5)
Supplement: Additional file 1: — Median values and quartiles (q1-q3) for determinants of the child’s environment and fruit/vegetable consumption. (DOCX 25 kb) [file 12889_2015_2540_MOESM1_ESM.docx]

**Additional file 1**. Median values and quartiles (q_1_-q_3_) for determinants of the child’s social environment and fruit/vegetable consumption.

|  | **Determinants of the social environment** | | | | | | | | | | | | | | | | | | | | | | | | | | | | **Determinants of the physical environment** | | | |
| --- | --- | --- | --- | --- | --- | --- | --- | --- | --- | --- | --- | --- | --- | --- | --- | --- | --- | --- | --- | --- | --- | --- | --- | --- | --- | --- | --- | --- | --- | --- | --- | --- |
|  | Parental Demand  *Never (0) - yes, always (4)* | | | | | Parental allowance  *Never (0) - yes, always (4)* | | | | | | Active encouragement  *fully disagree (-2 ) -fully agree (2)* | | | | | Facilitating  *Never (0) - yes, always (4)*  *(F18,F31)* | | | Parental knowledge on recommendations  *None (1) - 5 or more pieces/portions per day (8)* | | | | | Performing EBRB together with the child  *Never (0 ) - yes, always (4)* | | | | Home availability  *Never (0) - yes, always (4)* | | Habit to eat fruit/vegetables daily  *(-2) fully disagree-(2) fully agree* | |
| **Fruit consumption** | | | | | | | | | | | | | | | | | | | | | | | | | | | | | | | | |
| **Educational level (mother)**  **Country** | | High | | Low | High | | | Low | | | High | | | Low | High | | | | Low | | High | | Low | | High | | Low | | High | Low | High | Low |
| Belgium | | 3 (2-3) | | 3 (2-3) | 4 (3-4) | | | 4 (3-4) | | | 2 (1-2) | | | 2 (1-2) | 2 (1-3) | | | | 2 (1-3) | | 5 (4-6) | | 5 (4-6) | | 1 (0-2) | | 1 (0-1) | | **3 (3-4)**** | **3 (2-4)** | 0 (-1-2) | 0 (-1-1) |
| Bulgaria | | 4 (3-4) | | 4 (2-4) | **4 (4-4)**** | | | **4 (3-4)** | | | 2 (2-2) | | | 2 (2-2) | 3 (2-3) | | | | 3 (2-4) | | 6 (5-6) | | 6 (5-6) | | 2 (1-2) | | 2 (1-2) | | 3 (3-4) | 3 (2-4) | 1 (1-2) | 2 (1-2) |
| France | | 3 (2-4) | | 3 (2-3) | 3 (3-4) | | | 3 (3-4) | | | 1 (1-2) | | | 1 (1-2) | 2 (1-3) | | | | 2 (1-2) | | 5 (4-5) | | 5 (4-5) | | 1 (0-1) | | 1 (0-1) | | 3 (2-3) | 3 (2-3) | 1 (-1-2) | 1 (0-1) |
| Greece | | 3 (3-4) | | 4 (3-4) | 4 (4-4) | | | 4 (3-4) | | | 2 (1-2) | | | 2 (2-2) | 3 (2-4) | | | | 3 (2-4) | | 5 (5-6) | | 5 (5-6) | | 1 (1-2) | | 2 (1-2) | | 4 (3-4) | 3 (3-4) | **1 (0-1)*** | **1 (1-2)** |
| Portugal | | 4 (3-4) | | 4 (3-4) | 4 (3-4) | | | 3 (3-4) | | | 2 (2-2) | | | 2 (2-2) | 2 (2-3) | | | | 2 (2-3) | | 5 (5-6) | | 5 (5-6) | | **2 (2-2)**** | | **2 (1-2)** | | **4 (3-4)**** | **3 (3-4)** | 2 (1-2) | 2 (1-2) |
| Romania | | 4 (3-4) | | 4 (3-4) | 4 (4-4) | | | 4 (4-4) | | | 2 (2-2) | | | 2 (2-2) | **2 (2-3)*** | | | | **2 (2-3)** | | 5 (5-6) | | 5 (4-6) | | 2 (1-2) | | 2 (2-2) | | **4 (3-4)**** | **3 (2-4)** | 2 (1-2) | 2 (1-2) |
| The Netherlands | | **3 (3-4)***** | | **3 (2-3)** | 4 (3-4) | | | 4 (3-4) | | | 2 (1-2) | | | 2 (1-2) | 3 (2-3) | | | | 3 (2-3) | | 5 (5-5) | | 5 (4-5) | | 1 (1-2) | | 1 (0-2) | | 4 (3-4) | 4 (3-4) | 2 (1-2) | 1 (1-2) |
| Total | | **3 (3-4)**** | | **3 (3-4)** | **4 (3-4)***** | | | **4 (3-4)** | | | 2 (1-2) | | | 2 (1-2) | **2 (2-3)***** | | | | **2 (2-3)** | | 5 (5-6) | | 5 (4-6) | | 2 (1-2) | | 1 (1-2) | | **3 (3-4)***** | **3 (3-4)** | **2 (1-2)*** | **1 (0-2)** |
| **Vegetable consumption** | | | | | | | | | | | | | | | | | | | | | | | | | | | | | | | | |
| **Educational level (mother)**  **Country** | | | High | Low | | | High | | Low | High | | | Low | | | High | | Low | | | | High | | Low | | High | | Low | High | Low | High | Low |
| Belgium | | | 4 (3-4) | 3 (3-4) | | | 4 (3-4) | | 4 (3-4) | 2 (1-2) | | | 1 (1-2) | | | 3 (2-4) | | 3 (3-4) | | | | 5 (4-6) | | 5 (5-6) | | 2 (1-2) | | 1 (1-2) | 3 (3-4) | 3 (3-4) | 1 (0-2) | 1 (0-2) |
| Bulgaria | | | 4 (3-4) | 4 (3-4) | | | 4 (4-4) | | 4 (4-4) | 2 (1-2) | | | 2 (1-2) | | | 3 (3-4) | | 3 (2-4) | | | | 6 (5-6) | | 5 (4-6) | | 2 (1-2) | | 2 (1-2) | 3 (3-4) | 3 (3-4) | 1 (1-2) | 2 (0-2) |
| France | | | 4 (3-4) | 3 (3-4) | | | 3 (3-4) | | 3 (3-4) | 1 (1-2) | | | 1 (1-2) | | | 3 (3-4) | | 3 (3-3) | | | | 5 (4-6) | | 5 (4-6) | | 1 (1-2) | | 1 (0-1) | 3 (3-4) | 3 (3-3) | 1 (0-2) | 1 (0-1) |
| Greece | | | 3 (3-4) | 3 (3-4) | | | 4 (4-4) | | 4 (4-4) | 1 (1-2) | | | 2 (1-2) | | | 3 (3-4) | | 3 (3-4) | | | | **4 (4-5)*** | | **4 (4-5)** | | 1 (1-2) | | 2 (1-2) | 4 (3-4) | 3 (3-4) | 0 (0-1) | 1 (0-1) |
| Portugal | | | 4 (3-4) | 4 (3-4) | | | **4 (3-4)*** | | **3 (3-4)** | 2 (1-2) | | | 2 (1-2) | | | **3 (3-4)*** | | **3 (2-3)** | | | | **5 (5-6)***** | | **5 (4-6)** | | 2 (1-2) | | 2 (1-2) | **3 (3-4)**** | **3 (2-4)** | 2 (1-2) | 1 (1-2) |
| Romania | | | 4 (3-4) | 4 (3-4) | | | 4 (4-4) | | 4 (4-4) | 2 (2-2) | | | 2 (2-2) | | | **3 (3-4)**** | | **3 (2-3)** | | | | 5 (4-6) | | 4 (4-5) | | 2 (1-2) | | 2 (2-2) | **4 (3-4)**** | **3 (3-4)** | 2 (1-2) | 2 (1-2) |
| The Netherlands | | | 4 (3-4) | 3 (3-4) | | | 4 (3-4) | | 4 (3-4) | 2 (2-2) | | | 2 (1-2) | | | **4 (3-4)***** | | **3 (3-4)** | | | | 5 (5-5) | | 5 (4-5) | | **2 (2-2)*** | | **2 (2-2) ^b^** | 3 (3-4) | 3 (3-4) | **2 (1-2)*** | **1 (1-2)** |
| **Total** | | | 4 (3-4) | 3 (3-4) | | | **4 (3-4)***** | | **4 (3-4)** | **2 (1-2)*** | | | **2 (1-2)** | | | **3 (3-4)***** | | **3 (2-4)** | | | | **5 (5-6)***** | | **5 (4-6)** | | **2 (1-2)**** | | **2 (1-2) ^a^** | **3 (3-4)***** | **3 (3-4)** | **1 (1-2)*** | **1 (0-2)** |

Comparison between the educational groups of each country and the total sample with Mann-Whitney U test. Rounded values are presented.

**Additional file 1**. Median values and quartiles (q_1_-q_3_) for determinants of the child’s social environment and fruit/vegetable consumption *(continued)*.

*(continued)*

*,**,***: significant at .05, .01 and .001 respectively
