# Supplementary material for: Inequities in energy-balance related behaviours and family environmental determinants in European children: baseline results of the prospective EPHE evaluation study
Source: BMC Public Health. 2015 Dec 2;15:1203. doi: 10.1186/s12889-015-2540-5 (PMC4668694; doi:10.1186/s12889-015-2540-5)
Supplement: Additional file 2: — Median values and quartiles (q1-q3) for determinants of the child’s social environment and fruit juices consumption. (DOCX 21 kb) [file 12889_2015_2540_MOESM2_ESM.docx]

| **Additional file 2.** Median values and quartiles (q_1_-q_3_) for determinants of the child’s social environment and fruit juices consumption. | **Determinants for the social environment** | | | | | | | | | | | | | | | | | | | | | | | | | |  |  |
| --- | --- | --- | --- | --- | --- | --- | --- | --- | --- | --- | --- | --- | --- | --- | --- | --- | --- | --- | --- | --- | --- | --- | --- | --- | --- | --- | --- | --- |
|  | Paying attention/  monitoring  *never (0)-always (4)* | | | Parental allowance  *never (0)-always (4)* | | | Negotiating  *never (0)-always (4)* | | | Communicating health beliefs  *never (0)-always (4)* | | | Avoid negative modelling  *never (0)-always (4)* | | | | Parental self- efficacy to retain rules  *never (0)-always (4)* | | Rewarding/comforting practice  *never (0)-always (4)* | | Performing EBRB together with the child  *Never (1 ) - every day more than once a day (7)* | | | Nagging behaviour  *Never (0 )-yes, always (4)* | | | | |
| **Fruit juices consumption** | | | | | | | | | | | | | | | | | | | | | | | | | | |  |  |
| **Educational level (mother)**  **Country** | | High | Low | | High | Low | | High | Low | | High | Low | | High | Low | High | | Low | High | Low | | High | Low | | High | Low | |  |
| Belgium | | 3 (2-4) | 3 (2-4) | | 3 (2-4)  **2 (1-3)*** | 3 (2-4)  **3 (2-4)** | | 2 (0-3) | 1 (0-2) | | 0 (0-2)  0 (0-0) | 0 (0-2)  0 (0-1) | | 0 (0-0) | 0 (0-0) | 0 (0-0) | | 0 (0-1) | 0 (0-0) | 0 (0-0) | | 4 (2-6) | 4 (3-6) | | 0 (0-0) | 0 (0-0) | |  |
| Bulgaria | | 3 (2-4) | 4 (3-4) | | 3 (3-4)  3 (3-4) | 4 (3-4)  4 (2-4) | | 3 (1-3) | 3 (2-4) | | 2 (0-3)  0 (0-2) | 2 (0-3)  0 (0-2) | | 2 (0-3) | 2 (0-4) | 1 (0-2) | | 1 (0-2) | 0 (0-1) | 0 (0-2) | | 3 (3-3) | 3 (3-3) | | 1 (0-2) | 1 (0-2) | |  |
| France | | 3 (2-4) | 3 (2-4) | | 3 (2-3)  2 (1-3) | 2 (2-3)  2 (1-3) | | 2 (0-3) | 2 (0-3) | | 0 (0-2)  **0 (0-0)**** | 1 (0-2)  **0 (0-2)** | | 0 (0-0) | 0 (0-0) | **0 (0-0)*** | | **0 (0-2)** | 0 (0-0) | 0 (0-0) | | 4 (2-6) | 4 (3-6) | | **0 (0-0)**** | **0 (0-2)** | |  |
| Greece | | 4 (3-4) | 4 (3-4) | | 4 (3-4)  3 (2-4) | 3 (2-4)  3 (2-4) | | **2 (1-3)*** | **3 (2-4)** | | 1 (0-2)  0 (0-2) | 1 (0-3)  0 (0-2) | | 0 (0-2) | 0 (0-2) | 1 (0-2) | | 1 (0-2) | 0 (0-1) | 0 (0-1) | | 3 (2-4) | 3 (2-4) | | 0 (0-1) | 1 (0-1) | |  |
| Portugal | | 4 (3-4) | 4 (3-4) | | 2 (2-2)  2 (1-2) | 2 (2-3)  2 (1-2) | | 1 (0-3) | 2 (0-3) | | 2 (1-3)  **1 (0-2)*** | 2 (1-3)  **2 (1-3)** | | 1 (0-2) | 1 (0-3) | 0 (0-1) | | 0 (0-1) | **0 (0-1)**** | **0 (0-1)** | | 3 (2-4) | 3 (2-4) | | 0 (0-1) | 0 (0-1) | |  |
| Romania | | **3 (3-4)*** | **3 (2-4)** | | 3 (2-4)  3 (2-4) | 3 (2-4)  3 (1-4) | | 1 (0-3) | 1 (0-2) | | 2 (0-3)  0 (0-2) | 1 (0-3)  0 (0-2) | | 0 (0-2) | 0 (0-2) | **0 (0-1)*** | | **1 (0-2)** | 0 (0-0) | 0 (0-0) | | 3 (2-4) | 3 (2-4) | | 1 (0-2) | 1 (0-2) | |  |
| The Netherlands | | 3 (2-4) | 3 (2-4) | | 2 (2-3)  1 (0-2) | 2 (2-3)  2 (0-3) | | 2 (0-3) | 1 (0-3) | | 1 (0-2)  1 (0-2) | 2 (0-3)  1 (0-2) | | 1 (0-2) | 1 (0-2) | **0 (0-0)***** | | **0 (0-1)** | **0 (0-0)*** | **0 (0-1)** | | 3 (2-4) | 2 (2-4) | | **0 (0-0)*** | **0 (0-1)** | |  |
| **Total** | | 3 (2-4) | 3 (2-4) | | 3 (2-4)  2 (1-4) | 3 (2-4)  2 (1-3) | | 2 (0-3) | 2 (0-3) | | 1 (0-2)  **0 (0-2)**** | 1 (0-3)  **1 (0-2)** | | 0 (0-2) | 0 (0-2) | 0 (0-1) | | 0 (0-1) | **0 (0-0)**** | **0 (0-1)** | | **3 (2-4)**** | **3 (2-4) ^a^** | | **0 (0-1)*** | **0 (0-1) ^a^** | |  |
| Comparison between the educational groups of each country and the total sample with Mann-Whitney U test. Rounded values are presented.  *,**,***: significant at .05, .01 and .001 respectively  ^a^  Negligible differences in spread were found between the two socio-economic groups. | | | | | | | | | | | | | | | | | | | | | | | | | | | |  |
