# Supplementary material for: Inequities in energy-balance related behaviours and family environmental determinants in European children: baseline results of the prospective EPHE evaluation study
Source: BMC Public Health. 2015 Dec 2;15:1203. doi: 10.1186/s12889-015-2540-5 (PMC4668694; doi:10.1186/s12889-015-2540-5)
Supplement: Additional file 3: — Median values and quartiles (q1-q3) for determinants of the child’s social environment and soft drinks consumption. (DOCX 20 kb) [file 12889_2015_2540_MOESM3_ESM.docx]

| **Additional file 3.** Median values and quartiles (q_1_-q_3_) for determinants of the child’s social environment and soft drinks consumption*.* | **Determinants for the social environment** | | | | | | | | | | | | | | | | | | | | | | | | |  |
| --- | --- | --- | --- | --- | --- | --- | --- | --- | --- | --- | --- | --- | --- | --- | --- | --- | --- | --- | --- | --- | --- | --- | --- | --- | --- | --- |
|  | | Paying attention/  monitoring  *never (0)-always (4)* | | | Parental allowance  *never (0)-always (4)* | | | Communicating health beliefs  *never (0)-always (4)* | | | Avoid negative modelling  *never (0)-always (4)* | | | Parental self- efficacy to retain rules  *never (0)-always (4)* | | | Rewarding/comforting practice  *never (0)-always (4)* | | Performing EBRB together with the child  *Never (1 )- every day more than once a day (7)* | | | | Nagging behaviour  *Never (0 )-yes, always (4)* | | | |
| **Soft drinks consumption** | | | | | | | | | | | | | | | | | | | | | | | | | | |
| **Educational level (mother)**  **Country** | | | High | Low | | High | Low | | High | Low | | High | Low | High | Low | High | | Low | | High | Low | High | | | Low | |
| Belgium | | | 4 (3-4) | 4 (3-4) | | 2 (1-3)  0 (0-1) | 2 (1-2)  0 (0-2) | | 3 (3-4)  3 (1-4) | 3 (3-4)  3 (1-4) | | 0 (0-2) | 0 (0-2) | 0 (0-0) | 0 (0-1) | 0 (0-0) | | 0 (0-0) | | 3 (1-4) | 3 (1-5) | 0 (0-1) | | | 0 (0-1) | |
| Bulgaria | | | 4 (4-4) | 4 (3-4) | | 1 (1-2)  1 (0-2) | 2 (1-2)  1 (1-2) | | 4 (3-4)  3 (1-4) | 4 (3-4)  3 (1-4) | | 3 (2-4) | 3 (2-4) | 0 (0-2) | 0 (0-1) | 0 (0-0) | | 0 (0-0) | | 2 (1-3) | 2 (1-3) | 0 (0-1) | | | 1 (0-1) | |
| France | | | 4 (3-4) | 4 (3-4) | | 2 (1-2)  1 (0-2) | 2 (1-2)  1 (0-2) | | 3 (2-3)  1 (0-3) | 3 (2-3)  2 (0-3) | | 0 (0-1) | 0 (0-2) | 0 (0-0) | 0 (0-1) | 0 (0-0) | | 0 (0-0) | | 3 (2-5) | 3 (2-5) | **0 (0-0)*** | | **0 (0-1)** | | |
| Greece | | | 4 (4-4) | 4 (4-4) | | 1 (0-1)  0 (0-1) | 1 (0-1)  0 (0-1) | | 4 (3-4)  3 (2-4) | 4 (3-4)  4 (3-4) | | 3 (1-3) | 3 (1-4) | 0 (0-1) | 0 (0-1) | 0 (0-0) | | 0 (0-0) | | 1 (1-2) | 1 (1-2) | 0 (0-1) | | 0 (0-1) | | |
| Portugal | | | 4 (3-4) | 4 (4-4) | | **1 (1-2)***  1 (0-1) | **1 (1-2) ^a^**  1 (0-1) | | 3 (3-4)  **2 (1-3)**** | 3 (3-4)  **3 (1-4)** | | 1 (1-3) | 2 (1-3) | 0 (0-1) | 0 (0-1) | 0 (0-1) | | 0 (0-0) | | **2 (1-3)**** | **2 (2-3)** | **0 (0-1)*** | | **0 (0-1)** | | |
| Romania | | | 4 (4-4) | 4 (3-4) | | **1 (0-2)****  **0 (0-1)**** | **1 (1-2)**  **1 (0-2)** | | 4 (3-4)  3 (0-4) | 4 (3-4)  3 (1-4) | | **3 (2-4)*** | **2 (1-3)** | 0 (0-1) | 0 (0-1) | 0 (0-0) | | 0 (0-0) | | **2 (1-2)***** | **3 (2-3)** | 1 (0-2) | | 1 (0-2) | | |
| The Netherlands | | | 4 (3-4) | 3 (3-4) | | 2 (1-3)  0 (0-2) | 2 (1-3)  0 (0-2) | | 2 (1-3)  1 (0-2) | 2 (1-3)  2 (0-2) | | 1 (0-2) | 1 (0-2) | 0 (0-0) | 0 (0-1) | 0 (0-0) | | 0 (0-0) | | 2 (2-3) | 3 (1-4) | 0 (0-0) | | 0 (0-1) | | |
| **Total** | | | 4 (3-4) | 4 (3-4) | | **1 (1-2)****  **0 (0-1)*** | **1 (1-2)**  **1 (0-1)** | | 3 (3-4)  2 (1-4) | 3 (2-4)  3 (1-4) | | **2 (0-3)**** | **2 (0-3)** | 0 (0-1) | 0 (0-1) | 0 (0-0) | | 0 (0-0) | | **2 (1-3)***** | **2 (2-4)** | 0 (0-1) | | 0 (0-1) | | |

Comparison between the educational groups of each country and the total sample with Mann-Whitney U test. Rounded values are presented.

*,**,***: significant at .05, .01 and .001 respectively ^a^: Differences in spread showed that the high education group allowed soft drinks more frequently.
