# Supplementary material for: Inequities in energy-balance related behaviours and family environmental determinants in European children: baseline results of the prospective EPHE evaluation study
Source: BMC Public Health. 2015 Dec 2;15:1203. doi: 10.1186/s12889-015-2540-5 (PMC4668694; doi:10.1186/s12889-015-2540-5)
Supplement: Additional file 4: — Median values and quartiles (q1-q3) for determinants of the child’s social environment and screen exposure. (DOCX 27 kb) [file 12889_2015_2540_MOESM4_ESM.docx]

| **Additional file 4.** Median values and quartiles (q_1_-q_3_) for determinants of the child’s social environment and screen exposure. | **Determinants for the social environment** | | | | | | | | | | | | | | | | | | | | | | | | | | | | | | | | | | | | | | | | | | | | |  |  |
| --- | --- | --- | --- | --- | --- | --- | --- | --- | --- | --- | --- | --- | --- | --- | --- | --- | --- | --- | --- | --- | --- | --- | --- | --- | --- | --- | --- | --- | --- | --- | --- | --- | --- | --- | --- | --- | --- | --- | --- | --- | --- | --- | --- | --- | --- | --- | --- |
|  | Paying attention/  monitoring  *never (0)-always (4)* | | | | Parental allowance  *never (0)-always (4)* | | | | Negotiating  *never (0)-always (4)* | | | | | | Communicating health beliefs  *never (0)-always (4)* | | | | | Avoid negative modelling  *never (0)-always (4)* | | | | | Parental self- efficacy to retain rules  *never (0)-always (4)* | | | | | | Rewarding/comforting practice  *never (0)-always (4)* | | | | | | Performing EBRB together with the child  *Never (0)- every day (5)* | | | | Nagging behaviour  *Never (0)-yes, always (4)* | | | | |  |  |
| **Television exposure** | | | | | | | | | | | | | | | | | | | | | | | | | | | | | | | | | | | | | | | | | | | | | |  |  |
| **Educational level**  **(mother)**  **Country** | | High | Low | | High | | Low | | High | | | Low | | | High | | Low | | | | High | | Low | | | High | | Low | | High | | | | Low | | | High | | Low | | | High | | Low | |  |  |
| Belgium | | **3 (2-4)***** | **3 (1-4)** | | 2 (2-3)  2 (0-2) | | 3 (2-3)  2 (0-3) | | 2 (0-3) | | | 2 (0-3) | | | 3 (2-3)  0 (0-2) | | 2 (2-3)  0 (0-3) | | | | 0 (0-2) | | 0 (0-2) | | | 0 (0-0) | | 0 (0-1) | | 0 (0-1) | | | | 0 (0-1) | | | **2 (2-3)**** | | **3 (2-3)** | | | 0 (0-0) | | 0 (0-1) | |  |  |
| Bulgaria | | 4 (3-4) | 3 (3-4) | | 3 (2-3)  2 (2-3) | | 2 (2-3)  2 (2-3) | | 3 (3-4) | | | 3 (2-4) | | | 3 (3-4)  2 (0-3) | | 3 (3-3)  3 (1-3) | | | | 2 (1-3) | | 3 (1-3) | | | 1 (0-2) | | 2 (2-3) | | 1 (0-2) | | | | 0 (0-2) | | | 2 (2-3) | | 3 (2-3) | | | 1 (0-2) | | 1 (0-2) | |  |  |
| France | | 2 (1-3) | 2 (1-3) | | 3 (2-3)  2 (1-3) | | 3 (2-4)  2 (1-3) | | **1 (0-3)*** | | | **2 (0-3)** | | | 3 (2-4)  0 (0-2) | | 3 (2-3)  0 (0-2) | | | | 0 (0-1) | | 0 (0-2) | | | 0 (0-0) | | 0 (0-2) | | 0 (0-2) | | | | 0 (0-2) | | | **2 (2-3)**** | | **3 (2-3)** | | | 0 (0-1) | | 0 (0-2) | |  |  |
| Greece | | 4 (3-4) | 3 (3-4) | | 2 (2-3)  1 (1-2) | | 3 (2-3)  1 (0-2) | | 3 (2-4) | | | 3 (2-3) | | | 3 (3-4)  2 (0-3) | | 3 (3-4)  2 (0-3) | | | | 2 (1-3) | | 2 (1-3) | | | **1 (0-1)*** | | **1 (0-2)** | | 1 (0-2) | | | | 1 (0-2) | | | 2 (2-3) | | 3 (2-3) | | | 1 (0-2) | | 1 (0-2) | |  |  |
| Portugal | | 3 (3-4) | 3 (2-4) | | 2 (2-3)  **2 (1-2)*** | | 3 (2-3)  **2 (1-3)** | | 1 (0-3) | | | 1 (0-3) | | | 2 (2-3)  1 (0-2) | | 2 (2-3)  1 (0-2) | | | | 1 (0-2) | | 1 (0-2) | | | 0 (0-1) | | 1 (0-1) | | **1 (0-1)*** | | | | **1 (0-2)** | | | 2 (2-3) | | 2 (2-3) | | | **1 (0-1)*** | | **1 (0-2)** | |  |  |
| Romania | | 4 (3-4) | 3 (3-4) | | 2 (2-3)  **2 (1-2)*** | | 2 (2-3)  **2 (2-3)** | | **2 (1-3)*** | | | **2 (0-2)** | | | 3 (2-4)  1 (0-3) | | 3 (2-4)  2 (0-3) | | | | 1 (0-2) | | 1 (0-2) | | | 0 (0-1) | | 1 (0-2) | | 1 (0-1) | | | | 1 (0-2) | | | **2 (2-3)**** | | **2 (2-3)** | | | 2 (0-2) | | 2 (1-2) | |  |  |
| The Netherlands | | **3 (3-4)*** | **3 (2-3)** | | **2 (2-3)***  1 (0-2) | | **2 (2-3)**  1 (0-2) | | 3 (2-4) | | | 3 (2-3) | | | 2 (2-3)  2 (0-2) | | 2 (1-3)  2 (0-3) | | | | **2 (1-3)**** | | **1 (0-2)** | | | 0 (0-1) | | 0 (0-1) | | 1 (0-2) | | | | 0 (0-2) | | | 2 (2-3) | | 2 (2-3) | | | 0 (0-1) | | 0 (0-2) | |  |  |
| **Total** | | **3 (3-4)***** | **3 (2-4)** | | **2 (2-3)****  **2 (1-3)*** | | **3 (2-3)**  **2 (1-3) ^a^** | | **3 (1-3)***** | | | **2 (1-3)** | | | 3 (2-3)  1 (0-3) | | 3 (2-3)  1 (0-3) | | | | **1 (0-3)**** | | **1 (0-2)** | | | 0 (0-1) | | 0 (0-1) | | 1 (0-2) | | | | 1 (0-2) | | | **2 (2-3)***** | | **3 (2-3)** | | | **0 (0-2)*** | | **1 (0-2)** | |  |  |
| **Computer exposure** | | | | | | | | | | | | | | | | | | | | | | | | | | | | | | | | | | | | | | | | | | | | | |  |  |
| **Educational level (mother)**  **Country** | | High | | Low | | High | | Low | | | High | | | Low | | High | | Low | | | | High | | Low | | | High | | Low | | | High | | | Low | | | High | | Low | | | High | | Low | |  |
| Belgium | | 4 (3-4) | | 4 (2-4) | | 2 (1-2)  1 (0-2) | | 2 (1-2)  1 (0-2) | | | 2 (0-4) | | | 2 (0-3) | | 0 (0-2)  0 (0-2) | | 2 (2-3)  0 (0-3) | | | | 0 (0-2) | | 0 (0-2) | | | **0 (0-0)*** | | **0 (0-0)^b^** | | | 0 (0-1) | | | 0 (0-2) | | | **1 (0-2)*** | | **2 (0-2)** | | | **0 (0-0)*** | | **0 (0-0)** | |  |
| Bulgaria | | 4 (3-4) | | 4 (3-4) | | 2 (1-3)  2 (1-2) | | 2 (2-3)  2 (1-2) | | 3 (3-4) | | | 3 (2-4) | | | 3 (3-4)  2 (0-3) | | | 3 (3-3)  3 (1-3) | | | 2 (1-3) | | 2 (2-3) | | | 1 (0-2) | | 1 (0-2) | | | | 1 (0-2) | | | 0 (0-0) | | **1 (0-2)**** | | **2 (1-3)** | | | 0 (0-1) | | 0 (0-1) | | |
| France | | 3 (2-4) | | 3 (2-4) | | 2 (2-3)  1 (0-2) | | 2 (2-3)  1 (0-2) | | 1 (0-3) | | | 2 (0-3) | | | 3 (2-4)  0 (0-2) | | | 3 (2-3)  0 (0-2) | | | 0 (0-1) | | 0 (0-2) | | | 0 (0-0) | | 0 (0-0) | | | | 0 (0-1) | | | 0 (0-1) | | 2 (1-2) | | 1 (0-2) | | | **0 (0-0)*** | | **0 (0-1)** | | |
| Greece | | - | | - | | 2 (2-3)  1 (1-2) | | 2 (2-2)  1 (0-2) | | 3 (2-4) | | | 3 (2-3) | | | 3 (3-4)  2 (0-3) | | | 2 (1-3) | | | 2 (1-3) | | 2 (1-3) | | | 0 (0-1) | | 1 (0-1) | | | | 1 (0-2) | | | 1 (0-2) | | 1 (1-2) | | 1 (0-2) | | | 1 (0-2) | | 1 (0-2) | | |
| Portugal | | 3 (3-4) | | 4 (2-4) | | 2 (2-2)  1 (1-2) | | 2 (2-2)  2 (1-2) | | 2 (0-3) | | | 2 (0-3) | | | 2 (2-3)  1 (0-2) | | | 2 (2-3)  1 (0-2) | | | 1 (0-2) | | 1 (0-2) | | | 0 (0-1) | | 0 (0-1) | | | | 1 (0-1) | | | 1 (0-2) | | 2 (1-2) | | 2 (1-2) | | | 1 (0-1) | | 1 (0-1) | | |
| Romania | | 4 (3-4) | | 4 (3-4) | | 2 (2-3)  2 (1-2) | | 2 (2-3)  2 (1-2) | | **2 (0-3)*** | | | **2 (0-3)** | | | 3 (2-4)  1 (0-3) | | | 3 (3-4)  2 (0-3) | | | 1 (0-2) | | 1 (0-2) | | | 0 (0-1) | | 0 (0-1) | | | | 0 (0-1) | | | 0 (0-1) | | 2 (1-2) | | 2 (2-2) | | | 1 (0-2) | | 2 (0-2) | | |
| The Netherlands | | 3 (3-4) | | 3 (3-4) | | 2 (2-2)  1 (0-1) | | 2 (2-3)  1 (0-2) | | **3 (3-4)*** | | | **3 (2-4)** | | | 2 (2-3)  2 (0-2) | | | 2 (1-3)  2 (0-3) | | | **2 (1-3)*** | | **1 (0-2)** | | | 0 (0-1) | | 0 (0-1) | | | | 1 (0-2) | | | 0 (0-2) | | 2 (1-2) | | 2 (1-2) | | | 0 (0-1) | | 0 (0-1) | | |
| **Total** | | 3 (3-4) | | 4 (3-4) | | 2 (2-3)  1 (1-2) | | 2 (2-3)  1 (0-2) | | **3 (3-4)***** | | | **2 (0-3)** | | | 3 (2-3)  1 (0-3) | | | 3 (2-3)  1 (0-3) | | | **1 (0-3)**** | | **1 (0-2)** | | | 0 (0-1) | | 0 (0-1) | | | | 1 (0-2) | | | 0 (0-2) | | **2 (1-2)*** | | **2 (1-2) ^a^** | | | 0 (0-1) | | 0 (0-2) | | |

Comparison between the educational groups of each country and the total sample with Mann-Whitney U test. Rounded values are presented.

**Additional file 4**. Median values and quartiles (q_1_-q_3_) for determinants of the child’s social environment and screen exposure *(continued)*.

*(continued)*

*,**,***: significant at .05, .01 and .001 respectively

^a^: Negligible differences in spread were found between the socio-economic groups. ^b^: Differences in spread showed that the low education group had more often the self-efficacy to manage child’s computer exposure.
