# Supplementary material for: Inequities in energy-balance related behaviours and family environmental determinants in European children: baseline results of the prospective EPHE evaluation study
Source: BMC Public Health. 2015 Dec 2;15:1203. doi: 10.1186/s12889-015-2540-5 (PMC4668694; doi:10.1186/s12889-015-2540-5)
Supplement: Additional file 5: — Median values and quartiles (q1-q3) for determinants of the child’s physical environment and energy-balance related behaviours. (DOCX 34 kb) [file 12889_2015_2540_MOESM5_ESM.docx]

**Additional file 5.** Median values and quartiles (q_1_-q_3_) for determinants of the child’s physical environment and energy-balance related behaviours.

| **Determinant of physical environment** | | | | | **Country** | | | | | | | | | | | | | | | | | | | | | | | | | | | | | | | | | | | | | | | | | | | | | | | | | | | | | | | | | | | | | | | |
| --- | --- | --- | --- | --- | --- | --- | --- | --- | --- | --- | --- | --- | --- | --- | --- | --- | --- | --- | --- | --- | --- | --- | --- | --- | --- | --- | --- | --- | --- | --- | --- | --- | --- | --- | --- | --- | --- | --- | --- | --- | --- | --- | --- | --- | --- | --- | --- | --- | --- | --- | --- | --- | --- | --- | --- | --- | --- | --- | --- | --- | --- | --- | --- | --- | --- | --- | --- | --- |
|  | | **Belgium** | | | | | | | | | **Bulgaria** | | | | | | | | | | **France** | | | | | | | | | **Greece** | | | | | | | | | | | | **Portugal** | | | | | | **Romania** | | | | | | | | | | **The Netherlands** | | | | | **Total** | | | | | |
| **Water consumption** | | | | | | | | | | | | | | | | | | | | | | | | | | | | | | | | | | | | | | | | | | | | | | | | | | | | | | | | | | | | | | | | | | | | |
| **Educational level (mother)**  **Situation specific habit**  *(0)never- (4)always* | | High | | | | | Low | | | | High | | | | | Low | | | | | High | | | | Low | | | | | High | | | | | | Low | | | | | | High | | | | Low | | | | | High | | | | Low | | | | | High | | Low | | | High | Low | | |
| *During mealtime* | | **3 (2-4)**** | | | | | | **3 (1-4)** | | | 3 (2-3) | | | | | | | 3 (2-3) | | | 4 (3-4) | | | | | 3 (2-4) | | | | **3 (2-4)**** | | | | | | | **4 (3-4)** | | | | | 3 (3-4) | | | | 3 (3-4) | | | | | 2 (2-3) | | | | 2 (1-3) | | | | | 2 (1-4) | | 2 (1-3) | | | 3 (2-4) | | | 3 (2-4) |
| *Between meals* | | 3 (2-3) | | | | | | 3 (2-3) | | | 3 (3-4) | | | | | | | 4 (3-4) | | | 3 (2-4) | | | | | 3 (2-3) | | | | 3 (3-4) | | | | | | | 4 (3-4) | | | | | 3 (3-4) | | | | 4 (3-4) | | | | | 4 (3-4) | | | | 4 (3-4) | | | | | 2 (2-3) | | 2 (2-3) | | | 3 (2-4) | | | 3 (2-4) |
| *During/after sports/playing* | | 3 (3-4) | | | | | | 4 (3-4) | | | 4 (3-4) | | | | | | | 4 (3-4) | | | 4 (4-4) | | | | | 4 (3-4) | | | | 4 (4-4) | | | | | | | 4 (4-4) | | | | | 4 (3-4) | | | | 4 (3-4) | | | | | 4 (3-4) | | | | 4 (3-4) | | | | | **3 (2-3)*** | | **3 (2-4)** | | | 4 (3-4) | | | 4 (3-4) |
| **Fruit juices consumption** | | | | | | | | | | | | | | | | | | | | | | | | | | | | | | | | | | | | | | | | | | | | | | | | | | | | | | | | | | | | | | | | | | | | |
| **Educational level (mother)**  **1.Situation specific habit**  *yes (%)* | | | High | | | | | Low | | | High | | | | Low | | | | | | High | | | | Low | | | | | | High | | | | | | Low | | | | High | | | | Low | | | | | | High | | | | Low | | | | High | | | Low | | | High | | Low | |
| *During the weekend* | | | 35,5 | | | | | 37,3 | | | 42,4 | | | | 37,5 | | | | | | 42,0 | | | | 46,7 | | | | | | 44,0 | | | | | | 35,8 | | | | 57,8 | | | | 49,2 | | | | | | 44,1 | | | | 51,2 | | | | 27,1 | | | 38,5 | | | 43,7 | | 42,5 | |
| *At breakfast* | | | 56,6 | | | | | 45,1 | | | 15,8 | | | | 20,8 | | | | | | 59,8 | | | | 68,0 | | | | | | 22,7 | | | | | | 20,9 | | | | **9,2**** | | | | **1,6** | | | | | | 15,1 | | | | 7,5 | | | | 10,5 | | | 10,4 | | | 23,9 | | 24,4 | |
| *At lunch* | | | **7,9**** | | | | | **22,5** | | | 20,9 | | | | 20,8 | | | | | | 8,0 | | | | 13,0 | | | | | | 20,0 | | | | | | 23,9 | | | | 15,6 | | | | 10,2 | | | | | | 19,4 | | | | 27,5 | | | | 9,2 | | | 16,7 | | | 15,5 | | 18,4 | |
| *At dinner* | | | 10,5 | | | | | 17,6 | | | 29,5 | | | | 22,9 | | | | | | **4,0*** | | | | **18,5** | | | | | | 5,3 | | | | | | 1,5 | | | | 21,1 | | | | 29,7 | | | | | | 16,1 | | | | 7,5 | | | | 9,2 | | | 4,2 | | | 16,2 | | 16,5 | |
| *At school* | | | 56,6 | | | | | 59,8 | | | 23,7 | | | | 25,0 | | | | | | 6,0 | | | | 4,3 | | | | | | 30,7 | | | | | | 37,3 | | | | 28,4 | | | | 25,0 | | | | | | 4,3 | | | | 3,8 | | | | 18,4 | | | 33,3 | | | 24,4 | | 27,1 | |
| *While watching TV* | | | 6,6 | | | | | 14,7 | | | **10,1*** | | | | **22,9** | | | | | | 6,0 | | | | 14,1 | | | | | | 14,7 | | | | | | 10,4 | | | | **0,0*** | | | | **4,7** | | | | | | 6,5 | | | | 11,2 | | | | 7,9 | | | 18,8 | | | **7,3**** | | **12,4** | |
| *Between meals* | | | 15,8 | | | | | 26,5 | | | 24,5 | | | | 35,4 | | | | | | 16,0 | | | | 18,5 | | | | | | 42,7 | | | | | | 34,3 | | | | 1,8 | | | | 1,6 | | | | | | 25,8 | | | | 36,2 | | | | 17,1 | | | 16,7 | | | 20,2 | | 21,8 | |
| *During/after sports* | | | 6,6 | | | | | 14,7 | | | 9,4 | | | | 16,7 | | | | | | 2,0 | | | | 7,6 | | | | | | 26,7 | | | | | | 19,4 | | | | 1,8 | | | | 0,0 | | | | | | 3,2 | | | | 88 | | | | 6,6 | | | 8,3 | | | 7,9 | | 9,6 | |
| *At birthdays parties* | | | 48,7 | | | | | 38,2 | | | 49,6 | | | | 41,7 | | | | | | **56,0**** | | | | **77,2** | | | | | | 52,0 | | | | | | 44,8 | | | | 59,6 | | | | 54,7 | | | | | | **35,5*** | | | | **55,0** | | | | 27,6 | | | 31,2 | | | 47,8 | | 52,2 | |
| **2. Home availability**  *never (0)-always (4)* | | | 4 (3-4) | | | | | 4 (3-4) | | | 3 (2-3) | | | | 3 (2-3) | | | | | | 4 (3-4) | | | | 4 (3-4) | | | | | | 3 (2-4) | | | | | | 3 (2-4) | | | | 2 (1-3) | | | | 2 (2-3) | | | | | | 2 (2-3) | | | | 2 (2-3) | | | | 3 (2-4) | | | 3 (1-4) | | | 3 (2-4) | | 3 (2-4) | |
| **Soft drinks consumption** | | | | | | | | | | | | | | | | | | | | | | | | | | | | | | | | | | | | | | | | | | | | | | | | | | | | | | | | | | | | | | | | | | | | |
| **Educational level (mother)**  **1.Situation specific habit**  *yes (%)* | | | High | | | | | Low | | | | | High | | | | | | Low | | | | High | | | | | Low | | | High | | | Low | | | | | High | | | | | Low | | | | | High | | | | Low | | | | High | | | | Low | | | High | | | Low | |
| *During the weekend* | | | | 52,6 | | | | | 49,0 | | 28,8 | | | | | | | 33,3 | | | | 60,0 | | | | | 64,1 | | | | | 32,0 | | | 26,9 | | | | | 43,1 | | | | | 46,9 | | | | | 32,3 | | | | 46,2 | | | 36,8 | | | | 47,9 | | | **38,7**** | | | **46,5** | |
| *At breakfast* | | | | 0,0 | | | | | 1,0 | | 1,4 | | | | | | | 2,1 | | | | 0,0 | | | | | 2,2 | | | | | 0,0 | | | 0,0 | | | | | 0,0 | | | | | 0,8 | | | | | 0,0 | | | | 2,5 | | | 3,9 | | | | 8,3 | | | 0,8 | | | 1,9 | |
| *At lunch* | | | | 22,4 | | | | | 23,5 | | 7,9 | | | | | | | 8,3 | | | | 10,0 | | | | | 16,3 | | | | | 5,3 | | | 9,0 | | | | | **0,9*** | | | | | **7,8** | | | | | 5,4 | | | | 8,8 | | | 3,9 | | | | 4,2 | | | **7,4**** | | | **12,0** | |
| *At dinner* | | | | 22,4 | | | | | 24,5 | | 13,7 | | | | | | | 12,5 | | | | 14,0 | | | | | 17,4 | | | | | 0,0 | | | 1,3 | | | | | **10,1*** | | | | | **21,1** | | | | | 3,2 | | | | 6,2 | | | 10,5 | | | | 8,3 | | | **10,7*** | | | **14,7** | |
| *At school* | | | | 5,3 | | | | | 5,9 | | 12,2 | | | | | | | 22,9 | | | | 2,0 | | | | | 1,1 | | | | | 2,7 | | | 1,5 | | | | | 6,4 | | | | | 7,0 | | | | | 6,5 | | | | 5,0 | | | 25,0 | | | | 31,2 | | | 9,1 | | | 8,3 | |
| *While watching TV* | | | | 13,2 | | | | | 9,8 | | 6,5 | | | | | | | 14,6 | | | | 8,0 | | | | | 8,7 | | | | | 1,3 | | | 1,5 | | | | | 0,0 | | | | | 2,3 | | | | | 4,3 | | | | 7,5 | | | 14,9 | | | | 14,6 | | | 6,3 | | | 7,4 | |
| *Between meals* | | | | 21,1 | | | | | 17,6 | | 7,2 | | | | | | | 10,4 | | | | 16,0 | | | | | 27,2 | | | | | 5,3 | | | 3,0 | | | | | 0,0 | | | | | 2,3 | | | | | 6,5 | | | | 8,8 | | | 35,5 | | | | 22,9 | | | 11,5 | | | 12,6 | |
| *During/after sports* | | | | 3,9 | | | | | 10,8 | | 3,6 | | | | | | | 4,2 | | | | 0,0 | | | | | 1,1 | | | | | 0,0 | | | 3,0 | | | | | 0,0 | | | | | 0,8 | | | | | 3,2 | | | | **5,0** | | | **14,5*** | | | | **2,1** | | | 3,6 | | | 3,9 | |
| *At birthdays parties* | | | | 63,2 | | | | | 54,9 | | 59,7 | | | | | | | 62,5 | | | | 60,0 | | | | | 76,1 | | | | | 62,7 | | | 71,6 | | | | | 73,4 | | | | | 69,5 | | | | | 78,5 | | | | 75,0 | | | 51,3 | | | | 41,7 | | | 64,7 | | | 66,0 | |
| **2. Home availability**  *never (0)-always (4)* | | | | 2 (1-4) | | | | | 2 (1-4) | | | **1 (0-2)*** | | | | | **1 (0-2)** | | | | | 2 (2-4) | | | | | 2 (1-4) | | | | | 0 (0-1) | | | 0 (0-1) | | | | | **1 (1-2)**** | | | | | **2 (1-2)** | | | | | **1 (0-1)*** | | | | **1 (1-2)** | | | 2 (1-4) | | | | 2 (1-4) | | | **1 (0-2)***** | | | **2 (1-3)** | |
| **TV exposure** | | | | | | | | | | | | | | | | | | | | | | | | | | | | | | | | | | | | | | | | | | | | | | | | | | | | | | | | | | | | | | | | | | | | |
|  | High | | | | | Low | | | | High | | | | Low | | | | | | High | | | | Low | | | | | High | | | | Low | | | | | High | | | | | Low | | | | High | | | | | Low | | | | High | | | | | Low | | | High | | | Low | |
| **TV in bedroom**  *yes* (%) | **14,9%*** | | | | | **28,7%** | | | | **39,9%**** | | | | **66,0%** | | | | | | 20,0 % | | | | 31,1 % | | | | | 20,0% | | | | 20,9 % | | | | | **36,1%***** | | | | | **73,4 %** | | | | **48,9%***** | | | | | **75,3%** | | | | **5,3%***** | | | | | **31,3%** | | | **29,2%***** | | | **48,2%** | |
| **TV on during meal**  *(1) Every day- (5) Never* | **2 (1-5)*** | | | | | **1 (1-4)** | | | | 1 (1-3) | | | | 1 (1-3) | | | | | | 1 (1-1) | | | | 1 (1-1) | | | | | **4 (3-5)*** | | | | **4 (2-5)** | | | | | **1 (1-5)*** | | | | | **1 (1-4)#** | | | | 3 (1-6) | | | | | 3 (1-6)^ | | | | **5 (4-5)**** | | | | | **4 (3-5)** | | | **3 (1-5)***** | | | **1 (1-4)** | |

Rounded values are presented. Comparison of the educational groups per country, with Mann-Whitney U test for the categorical variables and Pearson’s Chi-square test for the binomial variables.

**Additional file 5.** Median values and quartiles (q_1_-q_3_) for determinants of the child’s physical environment and energy balance-related behaviours *(continued)*.

*,**,***: significant at .05, .01 and .001 respectively

#: Response categories by sequence (different from original [27]): 1. Every day 2. 4-6 days a week 3. 1-3 days a week 4. 2-4 days a week 5. Less than 1 day a week 6.Never

^: Response categories by sequence (different from original [27]): 1. Every day 2. 1-3 days a week 3. 2-4 days a week 4. 4-6 days a week 5. Less than 1 day a week 6.Never
