# Supplementary material for: Inequities in energy-balance related behaviours and family environmental determinants in European children: baseline results of the prospective EPHE evaluation study
Source: BMC Public Health. 2015 Dec 2;15:1203. doi: 10.1186/s12889-015-2540-5 (PMC4668694; doi:10.1186/s12889-015-2540-5)
Supplement: Additional file 6: — Corrected critical p -values after adjustment for multiple testing. (DOCX 28 kb) [file 12889_2015_2540_MOESM6_ESM.docx]

**Additional file 6**. Corrected critical p-values after adjustment for multiple testing.

| **Country** | **Belgium** | **Bulgaria** | **France** | **Greece** | **Portugal** | **Romania** | **Netherlands** | **TOTAL** |
| --- | --- | --- | --- | --- | --- | --- | --- | --- |
| **Corrected overall critical**  **p-value**  **Energy balance**  **related-behaviour** | **0.00210526** | **0.00105263** | **0.00052632** | **0.00053763** | **0.00842105** | **0.00421053** | **0.00315789** | **0.02157895** |
| Fruit consumption frequency (per week) | 0 | 0 | 0 | 0 | 1 | 0 | 1 | 1 |
| Salad or grated vegetables frequency (per week) | 0 | 0 | 0 | 0 | 1 | 0 | 0 | 1 |
| Raw vegetables frequency (per week) | 0 | 0 | 0 | 0 | 0 | 0 | 0 | 1 |
| Cooked Vegetables | 0 | 0 | 0 | 0 | 1 | 0 | 0 | 0 |
| Water frequency | 0 | 0 | 0 | 0 | 0 | 0 | 0 | 0 |
| Fruit juices frequency (per week) | 0 | 0 | 0 | 0 | 0 | 0 | 0 | 0 |
| Fruit juices' amount | 0 | 0 | 0 | 0 | 0 | 0 | 0 | 1 |
| Soft drinks frequency | 0 | 0 | 0 | 0 | 1 | 1 | 0 | 1 |
| Soft drinks amount | 0 | 0 | 0 | 0 | 0 | 1 | 0 | 1 |
| TV weekdays | 1 | 0 | 0 | 0 | 1 | 1 | 0 | 1 |
| TV weekend days | 0 | 0 | 0 | 0 | 0 | 0 | 0 | 1 |
| PC weekdays | 0 | 1 | 0 | 0 | 0 | 0 | 0 | 0 |
| PC weekend days | 0 | 0 | 0 | 0 | 0 | 0 | 0 | 0 |
| Total screen time | 1 | 0 | 0 | 0 | 1 | 0 | 0 | 1 |
| Sleep hours-Week days | 0 | 0 | 0 | 0 | 1 | 0 | 1 | 0 |
| Sleep hours-Weekend days | 0 | 0 | 0 | 0 | 0 | 0 | 0 | 0 |
| **Determinants of fruit consumption** | | | | | | | | |
| Parental knowledge on recommendations | 0 | 0 | 0 | 0 | 0 | 0 | 0 | 0 |
| Active encouragement | 0 | 0 | 0 | 0 | 0 | 0 | 0 | 0 |
| Performing EBRB together with the child | 0 | 0 | 0 | 0 | 1 | 0 | 0 | 0 |
| Habit to eat fruit daily | 0 | 0 | 0 | 0 | 0 | 0 | 0 | 0 |
| Parental demand | 0 | 0 | 0 | 0 | 0 | 0 | 1 | 1 |
| Parental allowance | 0 | 0 | 0 | 0 | 0 | 0 | 0 | 1 |
| Home availability | 0 | 0 | 0 | 0 | 1 | 0 | 0 | 1 |
| Parental facilitation | 0 | 0 | 0 | 0 | 0 | 0 | 0 | 1 |
| **Determinants of vegetable consumption** | | | | | | | | |
| Parental knowledge on recommendations | 0 | 0 | 0 | 0 | 1 | 0 | 0 | 1 |
| Active encouragement | 0 | 0 | 0 | 0 | 0 | 0 | 0 | 0 |
| Performing EBRB together with the child | 0 | 0 | 0 | 0 | 0 | 0 | 0 | 1 |
| Habit to eat vegetables daily | 0 | 0 | 0 | 0 | 0 | 0 | 0 | 1 |
| Parental demand | 0 | 0 | 0 | 0 | 0 | 0 | 0 | 0 |
| Parental allowance | 0 | 0 | 0 | 0 | 0 | 0 | 0 | 1 |
| Home availability | 0 | 0 | 0 | 0 | 1 | 0 | 0 | 1 |
| Parental facilitation | 0 | 0 | 0 | 0 | 0 | 0 | 1 | 1 |
| **Determinants of fruit juices consumption** | | | | | | | | |
| Home availability | 0 | 0 | 0 | 0 | 0 | 0 | 0 | 0 |
| Paying attention/monitoring | 0 | 0 | 0 | 0 | 0 | 0 | 0 | 0 |
| Parental allowance 1 | 0 | 0 | 0 | 0 | 0 | 0 | 0 | 0 |
| Parental allowance 2 | 0 | 0 | 0 | 0 | 0 | 0 | 0 | 0 |
| Negotiating | 0 | 0 | 0 | 0 | 0 | 0 | 0 | 0 |
| Communicating the health belief 1 | 0 | 0 | 0 | 0 | 0 | 0 | 0 | 0 |
| Communicating the health belief 2 | 0 | 0 | 0 | 0 | 0 | 0 | 0 | 1 |
| Avoid negative modelling | 0 | 0 | 0 | 0 | 0 | 0 | 0 | 0 |
| Nagging behaviour | 0 | 0 | 0 | 0 | 0 | 0 | 0 | 0 |
| Parental self- efficacy to retain rules | 0 | 0 | 0 | 0 | 0 | 0 | 0 | 0 |
| Rewarding/comforting practice | 0 | 0 | 0 | 0 | 1 | 0 | 0 | 1 |
| Conducting energy-balance related behaviour together with the child | 0 | 0 | 0 | 0 | 0 | 0 | 0 | 1 |
| **Determinants of soft drinks consumption** | | | | | | | | |
| Home availability | 0 | 0 | 0 | 0 | 1 | 1 | 0 | 1 |
| Paying attention/monitoring | 0 | 0 | 0 | 0 | 0 | 0 | 0 | 0 |
| Parental allowance 1 | 0 | 0 | 0 | 0 | 0 | 1 | 0 | 1 |
| Parental allowance 2 | 0 | 0 | 0 | 0 | 0 | 0 | 0 | 0 |
| Communicating health belief 1 | 0 | 0 | 0 | 0 | 0 | 0 | 0 | 0 |
| Communicating health belief 2 | 0 | 0 | 0 | 0 | 1 | 0 | 0 | 0 |
| Avoid negative modelling | 0 | 0 | 0 | 0 | 0 | 0 | 0 | 1 |
| Nagging behaviour | 0 | 0 | 0 | 0 | 0 | 0 | 0 | 0 |
| Parental self- efficacy to retain rules | 0 | 0 | 0 | 0 | 0 | 0 | 0 | 0 |
| Rewarding/comforting practice | 0 | 0 | 0 | 0 | 0 | 0 | 0 | 0 |
| Conducting energy-balance related behaviour together with the child | 0 | 0 | 0 | 0 | 0 | 1 | 0 | 1 |
| **Determinants of television exposure** | | | | | | | | |
| TV in child’s bedroom | 0 | 0 | 0 | 0 | 1 | 1 | 1 | 1 |
| TV on during meal time | 0 | 0 | 0 | 0 | 0 | 0 | 0 | 1 |
| Paying attention/monitoring | 1 | 0 | 0 | 0 | 0 | 0 | 0 | 1 |
| Parental allowance 1 | 0 | 0 | 0 | 0 | 0 | 0 | 0 | 1 |
| Parental allowance 2 | 0 | 0 | 0 | 0 | 0 | 0 | 0 | 1 |
| Negotiating | 0 | 0 | 0 | 0 | 0 | 0 | 0 | 1 |
| Avoid negative modelling | 0 | 0 | 0 | 0 | 0 | 0 | 1 | 1 |
| Nagging behaviour | 0 | 0 | 0 | 0 | 0 | 0 | 0 | 1 |
| Parental self- efficacy to retain rules | 0 | 0 | 0 | 0 | 0 | 0 | 0 | 0 |
| Rewarding/comforting practice | 0 | 0 | 0 | 0 | 0 | 0 | 0 | 0 |
| Performing energy-balance related behaviour together with the child | 1 | 0 | 0 | 0 | 0 | 1 | 0 | 1 |
| Communicating health belief 1 (TV and PC exposure) | 0 | 0 | 0 | 0 | 0 | 0 | 0 | 0 |
| Communicating health belief 2 (TV and PC exposure) | 0 | 0 | 0 | 0 | 0 | 0 | 0 | 0 |
| **Determinants of computer exposure** | | | | | | | | |
| Paying attention/monitoring | 0 | 0 | 0 | 0 | 0 | 0 | 0 | 0 |
| Parental allowance 1 | 0 | 0 | 0 | 0 | 0 | 0 | 0 | 0 |
| Parental allowance 2 | 0 | 0 | 0 | 0 | 0 | 0 | 0 | 0 |
| Negotiating | 0 | 0 | 0 | 0 | 0 | 0 | 0 | 1 |
| Avoid negative modelling | 0 | 0 | 0 | 0 | 0 | 0 | 0 | 1 |
| Nagging behaviour | 0 | 0 | 0 | 0 | 0 | 0 | 0 | 0 |
| Parental self- efficacy to retain rules | 0 | 0 | 0 | 0 | 0 | 0 | 0 | 0 |
| Rewarding/comforting practice | 0 | 0 | 0 | 0 | 0 | 0 | 0 | 0 |
| Performing energy-balance related behaviour together with the child | 0 | 1 | 0 | 0 | 0 | 0 | 0 | 1 |
| **Situation specific habit for soft drinks consumption** | | | | | | | | |
| During the weekend | 0 | 0 | 0 | 0 | 0 | 0 | 0 | 1 |
| Breakfast | 0 | 0 | 0 | 0 | 0 | 0 | 0 | 0 |
| At Lunch | 0 | 0 | 0 | 0 | 0 | 0 | 0 | 1 |
| At Dinner | 0 | 0 | 0 | 0 | 0 | 0 | 0 | 0 |
| At school | 0 | 0 | 0 | 0 | 0 | 0 | 0 | 0 |
| While watching TV | 0 | 0 | 0 | 0 | 0 | 0 | 0 | 0 |
| As a thirst quencher between me | 0 | 0 | 0 | 0 | 0 | 0 | 0 | 0 |
| During\after sports | 0 | 0 | 0 | 0 | 0 | 0 | 0 | 0 |
| At birthdays\parties | 0 | 0 | 0 | 0 | 0 | 0 | 0 | 0 |
| **Situation specific habit for fruit juices consumption** | | | | | | | | |
| During the weekend | 0 | 0 | 0 | 0 | 0 | 0 | 0 | 0 |
| Breakfast | 0 | 0 | 0 | 0 | 1 | 0 | 0 | 0 |
| At Lunch | 0 | 0 | 0 | 0 | 0 | 0 | 0 | 0 |
| At Dinner | 0 | 0 | 0 | 0 | 0 | 0 | 0 | 0 |
| At school | 0 | 0 | 0 | 0 | 0 | 0 | 0 | 0 |
| While watching TV | 0 | 0 | 0 | 0 | 0 | 0 | 0 | 1 |
| As a thirst quencher between me | 0 | 0 | 0 | 0 | 0 | 0 | 0 | 0 |
| During\after sports | 0 | 0 | 0 | 0 | 0 | 0 | 0 | 0 |
| At birthdays\parties | 0 | 0 | 0 | 0 | 0 | 0 | 0 | 0 |

Multiple testing adjustment by the Benjamini and Hocheberg method.

**0=**the adjusted p-value is higher than the corrected critical p-value. **1=** the adjusted p-value is lower than the corrected critical p-value.
